# The Hybrid Aesthetic Functional (HAF) Appliance: A Less Visible Proposal for Functional Orthodontics

**DOI:** 10.1155/2013/298671

**Published:** 2013-07-17

**Authors:** Christos Livas

**Affiliations:** Department of Orthodontics, University of Groningen, University Medical Center Groningen, Hanzeplein 1. Postbus 30.001, 9700 RB Groningen, The Netherlands

## Abstract

In modern orthodontics, aesthetics appear to have a decisive influence on orthodontic appliance preferences and acceptability. This paper reports the early application of a newly emerged functional device with enhanced aesthetics in a Class II treatment. Patient perspectives and technical considerations are discussed along with recommendations for further design development. It can be assumed that the use of thermoplastic material-based appliances may meet both the therapeutic and aesthetic demands of young age groups.

## 1. Introduction

Contemporary orthodontic technology is undoubtedly driven by the growing interest of the public for aesthetically advanced treatment options. Reflecting this trend, alternative orthodontic systems, such as clear aligners and lingual appliances, have been perceived by patients as the most appealing in relation to different types of buccal fixed attachments [1, 2]. Acceptability and attractiveness of vacuum formed aligners were rated also high in children and adolescents and showed an increase by age evolution pattern, resembling the conclusions of adult studies [3]. Moreover, appliance nonvisibility has been attributed greater intellectual ability, a finding which, even in its extremity, may indicate the impact of orthodontic appliance design on social perceptions [2]. Clinicians are nowadays enabled to select from a wide variety of clear tray trademarks that guide gradual tooth movement by means of a series of custom-made removable devices to satisfy patient's aesthetic demands with respect to appliance appearance. 

To our knowledge, up to this date, thermoplastic materials have not been engaged in the construction of functional orthopaedic devices. This article describes the design and clinical application of the prototype of the novel HAF appliance, a concept, which may bridge the gap between aesthetic preferences of young patients and hitherto dentofacial orthopaedics.

## 2. Appliance Design and Fabrication

**Figure 1:**
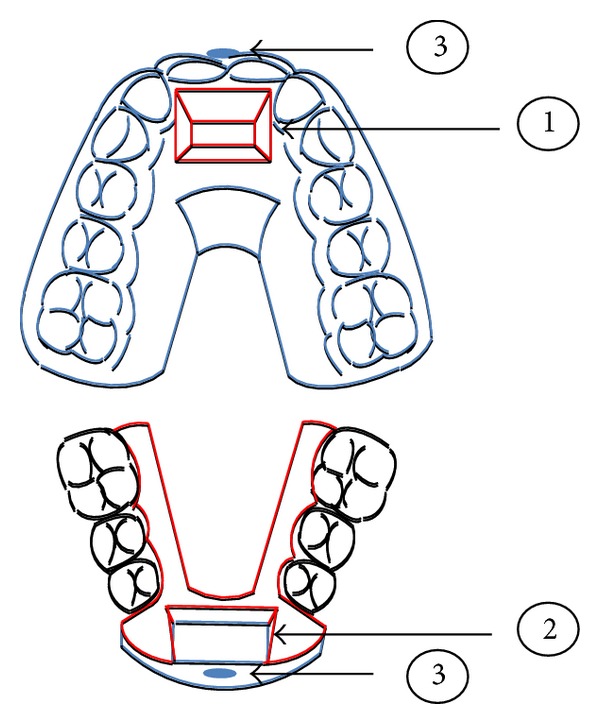
Schematic diagram of HAF appliance design. (1) Advancement bar. (2) Guiding surface. (3) Buttons (in blue ink: thermoplastic parts, in red ink: acrylic parts).

As the title implies, HAF appliance is intended to integrate aesthetics in functional orthodontic therapy. It is of double plate design, consisting of thermoplastic and acrylic parts (Figure 1). The upper component is a vacuum formed plate, made of transparent hard-elastic polyethylene sheet with 2 mm-thickness, equipped with an acrylic advancement bar positioned in the anterior midpalatal region. Similarly, the lower plate is constructed by thermoplastic material of the same width that covers totally the six anterior teeth. An acrylic guidance surface is placed over the plastic coverage on the lingual surfaces of lower incisors and canines, aimed to fit the upper bar. Two-sided arms are extended from the acrylic resin body up to second molars adapted to the morphology of lingual vestibule. To obtain optimal shape matching of the opposing attachments, the working casts are mounted on a semiadjustable articulator to contact in an end-to-end relationship, previously clinically registered with a construction bite. Posterior teeth are intentionally exposed to facilitate eruption and leveling of a deep curve of Spee. White colored acrylic resin is used for the fabrication of acrylic parts to enhance esthetics. By means of a special plier, buttons are formed at the cervical third of upper and lower dental midlines to enable night-time wear of vertical elastics. Alternatively, light-cured composite resin combined with appropriate transparent matrices can be used for button construction.

## 3. Case

### 3.1. Diagnosis and Treatment Plan

**Figure 2:**
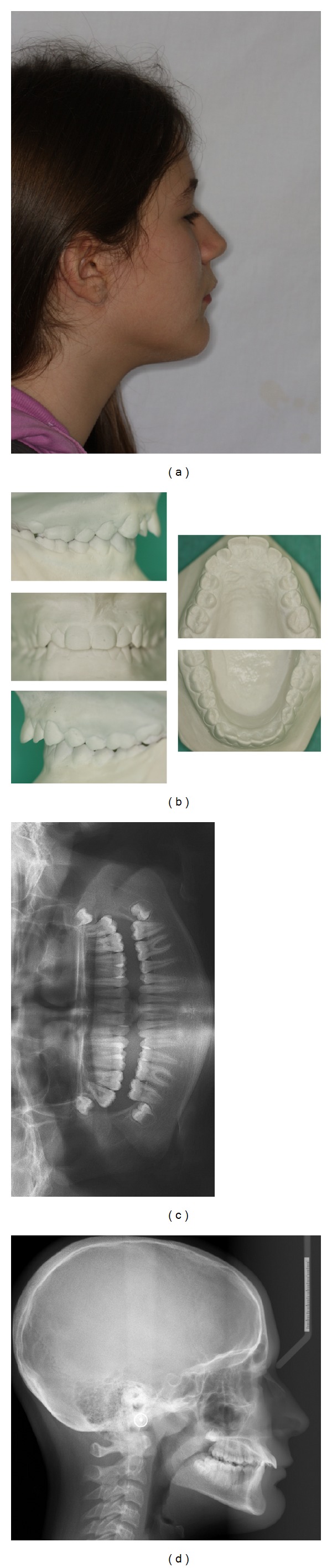
Pretreatment records: (a) extraoral profile photo, (b) dental casts, (c) panoramic radiograph, and (d) cephalometric radiograph.

An 11.6-year-old female was diagnosed with bilateral full cusp width Class II relationship, overjet of 11 mm, overbite of 5 mm, and mild crowded dental arches (Figure 2). Extraorally, the patient presented a convex profile with slightly prominent chin. Radiographic examination revealed signs of external apical root resorption for maxillary incisors, probably due to past occlusal trauma during bruxism activity. The treatment plan included two phases, an initial functional appliance stage for sagittal correction through mandibular advancement, following by limited full fixed mechanics to establish favorable tooth alignment and intercuspation. Appointments were determined on 4-week intervals, as generally suggested for compliance-dependent appliances [4].

### 3.2. Treatment Progress and Results

**Figure 3:**
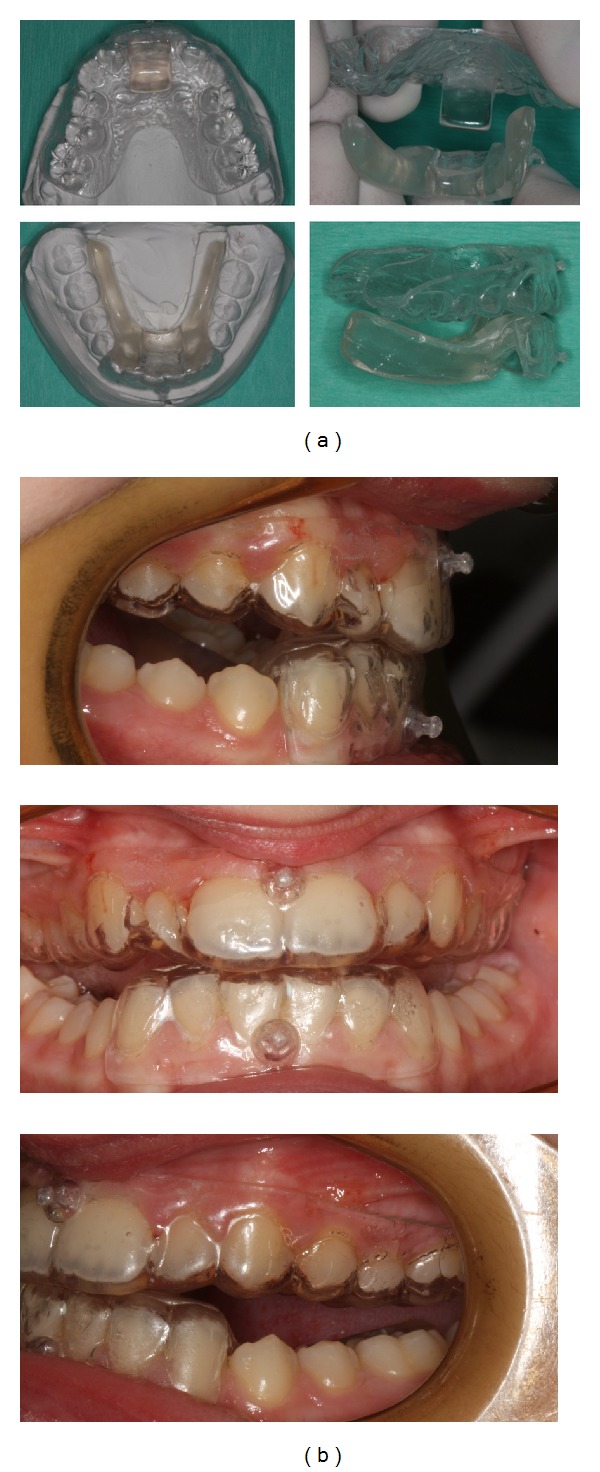
(a) Extraoral and (b) intraoral views of the HAF appliance used in this treatment.

Thorough instructions were given to the patient and her parents for appliance insertion, removal, and maintenance (Figure 3). The patient was tutored in front of a mirror to advance her mandible while closing the mouth in order to achieve matching of the acrylic surfaces. A minimum of 14 hours including bed time was prescribed for appliance wearing. Vertical intermaxillary elastic use was advised during sleeping to ensure mouth closure in the desired end-to-end relationship. Finally, the patient was asked to record herself the daily appliance wear, which was checked by the specialist at the beginning of each session.

**Figure 4:**
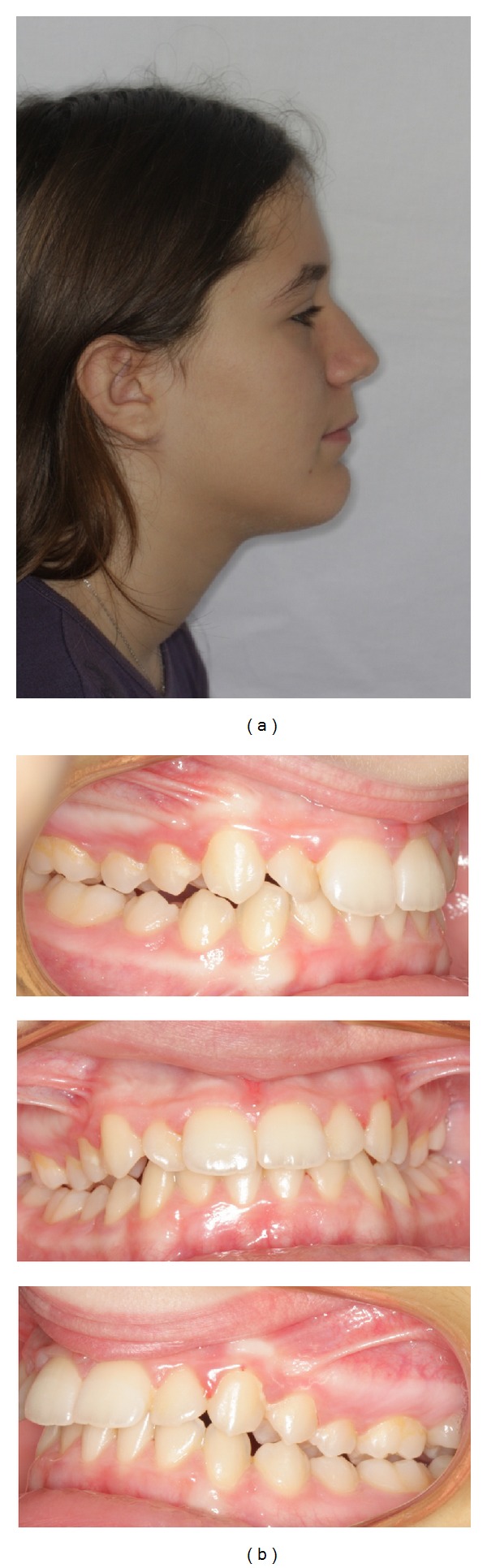
Intermediate records: (a) extraoral profile photo and (b) intraoral photos.

**Figure 5:**
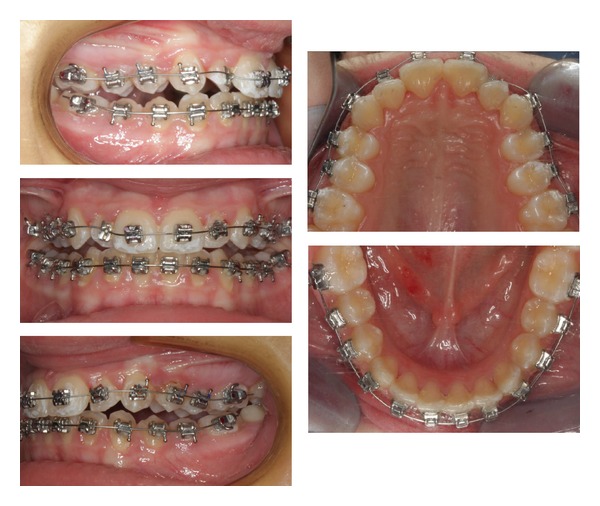
Intraoral photos with fixed appliances placed on both arches after a 10-month period in treatment.

**Figure 6:**
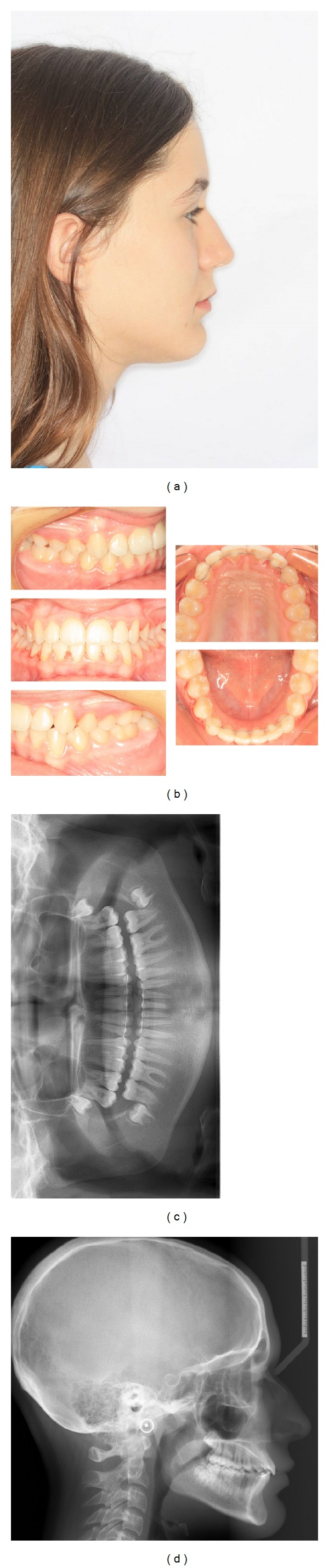
Posttreatment records: (a) extraoral profile photo, (b) dental casts, (c) panoramic radiograph, and (d) cephalometric radiograph.

Class I molar relationship was reached within eight months (Figure 4). Immediately after, Quick System (Forestadent, Pforzheim, Germany; http://www.forestadent.de/) self-ligating brackets (.022′′ slots, Roth prescription) were bonded in the mandibular arch, while the upper plate served shortly as anterior bite plane. Eight weeks later, fixed appliances were also placed on maxillary teeth, from first molar to first molar (Figure 5). Light force application was practised throughout fixed appliance phase to prevent aggravation of root resorption [5]. Without persisting on detailed root positioning, appliances were debonded after seven months (Figure 6). Pretreatment overjet and overbite values were reduced to 4 and 2 mm, respectively. In total, the two-stage treatment lasted seventeen months. Canine-to-canine bonded flexible spiral wire lingual retainers were placed on both arches for permanent retention. Additionally, an upper Hawley type retainer with anterior bite plane was prescribed for night-time wear aiming to prevent deep bite relapse. 

## 4. Discussion

**Figure 7:**
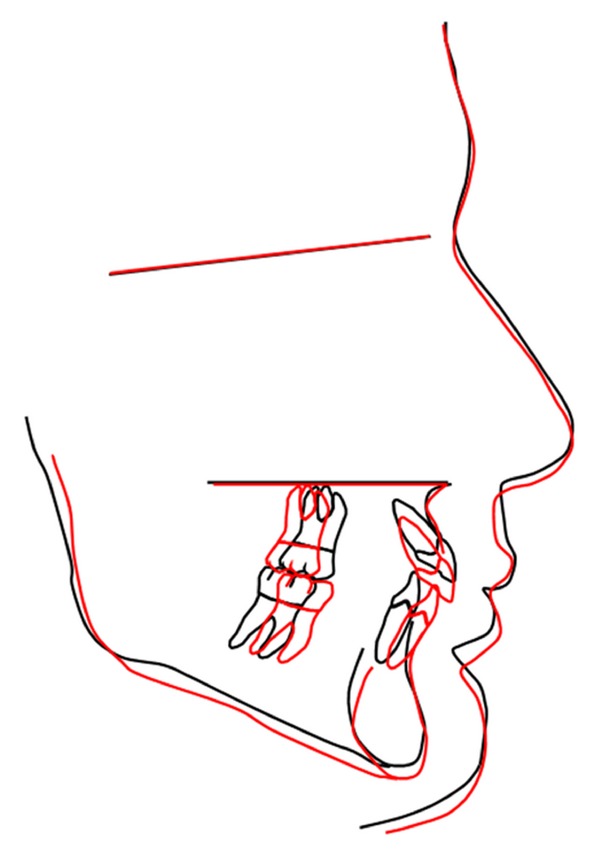
Superimposition of pre- and posttreatment cephalometric tracings.

This case report has demonstrated a successful 2-phase treatment of a Class II division 1 malocclusion including sequential use of a new “jumping the bite” device and fixed orthodontics. As indicated by the superimposition of the pre- and posttreatment cephalometric radiographs (Figure 7), the initial anteroposterior discrepancy has been corrected by treatment and growth associated effects, namely, forward and downward translation of the mandible in combination with reciprocal and opposite in direction maxillary and mandibular incisor tooth movement. Randomized clinical trials that investigated potential skeletal outcomes of various functional appliances [6–8] demonstrated small but statistically significant differences in mandibular length and especially higher effectiveness of “jumping the bite” appliances such as Herbst and Twin-Block compared to Andresen activator and related passive devices [9].

We were motivated to devise an aesthetically attractive removable appliance with complete lack of metal parts, largely transparent appearance, diminished bulkiness to avoid patient discomfort, and relatively less demanding laboratory fabrication. Ziuchkovski et al. [1] concluded that appliance attractiveness is inversely affected by the amount of visibility of metal parts. Likewise, best accepted functional appliances have been found to be the ones with reduced acrylic coverage occupying little space intraorally. On the contrary, appliances of design and shape causing excessive interocclusal opening with subsequent soft tissue tension and lip incompetence or constraining the tongue were the least approved [10].

Patient compliance manifested as sufficient wear time of the removable appliance is a major prerequisite for treatment success. Wear time prescription usually ranges from 13 to 16 hours per day and derives from empirical, nonevidence-based data owing to lack of objective measures and documentation. However, when it comes to real life, 8 out of 10 children may appear unwilling to extend plate use beyond night span [11]. Under such conditions, the actual wear time might decline to 9.5 hours daily and jeopardize a successful therapeutic outcome. According to the case records, the patient appeared to wear the appliance for approximately 12.5 each day, which practically proved to be sufficient to fulfill treatment requirements. Interestingly, she found it difficult to keep the intermaxillary elastics in place while sleeping and eventually abandoned elastic use. From a technical perspective, this fact questions the number and location of buttons, or even the presence of this feature per se in prospective versions. Modifications such as extension of the plastic coverage of the lower plate in favour of retention, radical reduction of the acrylic resin, and revision of matching of anterior attachments may be as well considered. 

Our initiative was to demonstrate one of our first attempts to produce a less visible and patient friendly functional device. Case reports inherently do not add rigorous scientific evidence. Thus, definite statements on the clinical performance of HAF appliance cannot be made. Overall, the development of a new appliance concept is a time-consuming process that necessitates meticulous observation and respective structural adjustment. Albeit the limited impact of our case presentation and early developmental stage of the device, HAF appliance treatment appeared to be beneficial and may deserve further research attention. 

 To conclude, using subsequently a novel functional device and fixed appliances, the sagittal skeletal discrepancy of a young female was corrected. Auxiliary development and clinical testing are required to augment the technical features and elucidate the treatment efficiency and effects of HAF appliance. On the whole, thermoplastic materials may be potentially utilized in the fabrication of functional orthodontic appliances.
